# Influence of PEA on Volume Stability of Cement-Based Grouting Materials and Its Mechanism

**DOI:** 10.3390/ma18040749

**Published:** 2025-02-08

**Authors:** Zheng Che, Tian-Liang Wang, Zheng-Guo Zhou, Shuo Wang, Xin-Wei Ma

**Affiliations:** 1 China Construction Sixth Engineering Bureau Hydropower Construction Co., Ltd., Tianjin 300222, China; chezheng0314@163.com (Z.C.); wangtianliang2024@163.com (T.-L.W.); zzg111121@163.com (Z.-G.Z.); 2 China Construction Sixth Engineering Bureau Co., Ltd., Tianjin 300450, China; 3 School of Ocean Engineering, Harbin Institute of Technology, Weihai 264209, China

**Keywords:** cement-based grouting materials, plastic expansive agent (PEA), volume stability, bubble spacing coefficient

## Abstract

Traditional expansive agents often fail to address early-stage cracking issues of grouting. A plastic expansive agent (PEA) can generate evenly distributed and closely packed microbubbles in the cement-basted grouting materials during the grout’s setting process to cause volume expansion. However, its expansion mechanism is still unclear, and this restricts its practical application in engineering. Thus, the effects of PEA on the volume stability of grouting were evaluated in this research, and its mechanism was analyzed by setting time, pH, compressive strength, and bubble spacing coefficient. The results indicated that an increase in PEA content enhanced the volume expansion rate of the grout, while the bubble spacing coefficient gradually decreased and air contents increased. However, it was not advisable to blindly increase its contents. A higher content could lead to a less dense pore structure and a decrease in compressive strength. Therefore, the optimal content for PEA was approximately between 0.04% and 0.06%. Additionally, the expansion process of PEA was related to cement hydration. Therefore, by adjusting the mixture proportion, its expansion process could be designed to exhibit microexpansion properties.

## 1. Introduction

Cement-based grouting materials are widely used due to their high strength, versatility, and superior cost-effectiveness [1]. However, with the increasing diversification and complexity of grouting applications, there is a growing requirement for the durability of cement-based grouting materials [2]. Currently, the serious durability issues encountered in grouts primarily involve cracking, especially early-age cracking [3,4].

The shrinkage of cement-based grouting materials often significantly affects the bond strength and ion penetration of the grouting position [5]. Unlike concrete, the early shrinkage of grouts is relatively higher because it does not contain coarse aggregates. Therefore, in addition to exhibiting microexpansion properties during the later stage of hardening to compensate for the chemical shrinkage of the grout, it is even more crucial for it to display this capability in the early stage. This is to compensate for the plastic and autogenous shrinkage experienced by the grout with a low water–binder ratio and high fluidity, transitioning from a fresh state to a hardened state [6,7]. The volume stability of cement-based grouting materials has emerged as a critical scientific issue that urgently needs to be addressed.

Three mechanisms contribute to reducing shrinkage or limiting the cracking of grouts, namely the application of supplementary cementitious materials [8,9], the application of expansion agents [10,11], and the incorporation of fibers [12]. To mitigate the shrinkage of grout, commercial expansion agents such as calcium sulphoaluminate, calcium oxide, and magnesium oxide are now widely used [10,13]. These agents are widely employed to create non-shrinkage grouting materials, effectively addressing and eliminating cracking. However, research has indicated that the initial expansion effect of these agents is limited. Grouts containing these agents often fail to expand due to inadequate curing. Furthermore, the delayed expansion due to their sluggish hydration process may lead to the cracking of hardened grouts and diminish their durability [14,15,16].

According to reports, certain chemical substances, such as hydrazine homologs and azodicarbonamide, have been widely used as blowing agents in the production of porous materials [17]. Their influence is based on the theory that their thermal decomposition leads to the release of N_2_, CO, CO_2_, and NH_3_ [18]. Recent studies have shown that, when utilized in cement-based grouting materials, they exhibit the characteristic of decomposing in strongly alkaline environments. The decomposition reaction produces gas, which manifests as tiny bubbles evenly distributed within the grout, resembling the type of bubbles introduced by air-entraining agents [19]. This type of reaction occurs when the grout is still in its plastic state, so this substance is referred to as a plastic expansion agent (PEA). Therefore, PEA is an emerging solution for addressing early cracking issues in grouting. The chemical reaction equation is presented as follows [20]:

H_2_N-NH_2_ + 4OH^−^ − 4e ⟶ N_2_↑ + 4H_2_O (1)

C_2_H_4_N_4_O_2_ + 2OH^−^ ⟶ N_2_C_2_O_4_^2−^ + 2NH_3_↑ (2)

2N_2_C_2_O_4_^2−^ + 2H_2_O ⟶ N_2_↑ + 2CO_2_↑ + N_2_H_4_ + 2CO_3_^2−^ (3)

The formation of microbubbles increases the volume of the grout, which can effectively compensate for the shrinkage of the grout and even induce slight expansion. However, the current studies do not clearly comprehend the expansion mechanism of PEA. PEA only plays a role in the early stage of the grout’s shrinkage [21,22]. The window period during which PEA exerts its effect has not been adequately captured. In the field of grouting technology, there is a relative scarcity of relevant literature. Therefore, gaining a deep understanding of its expansion mechanism is crucial to fully harnessing its potential. Furthermore, high-performance cement-based grouting materials typically contain a significant amount of supplementary cementitious materials, including fly ash [23], ground granulated blast furnace slag [24], and silica fume [25]. These materials have a significant impact on the hydration process of fresh grout, thereby influencing its fluidity, viscosity, and strength development process [26,27]. The effects of mixture proportions on PEA are far from being clearly understood, which restricts the practical application of PEA in engineering. The occurrence of the decomposition reaction of PEA either too early or too late is not conducive to the generation of expansion.

Therefore, the purpose of this paper is to study the mechanism through which the plastic expansive agent (PEA) functions against cracking. The volumetric measurement for grouts was utilized to assess the expansion effect of PEA. The impact of mixture proportions on PEA’s expansion progress was examined in conjunction with the setting time and pH. Additionally, the effects of PEA on compressive strength were explored. Lastly, the relationship between PEA’s cracking resistance and air-void parameters was investigated. The findings of this study provide a theoretical foundation for the effective expansion of PEA in grouting, thereby facilitating its widespread use.

## 2. Materials and Methods

### 2.1. Materials

**Figure 1:**
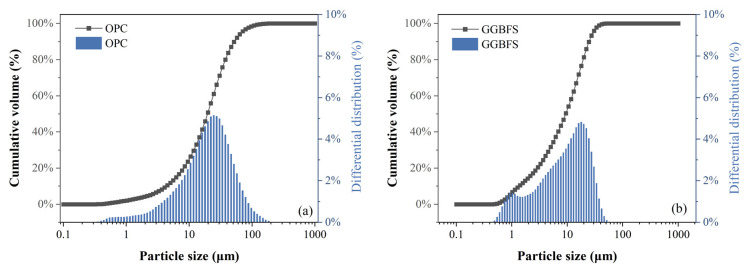
Particle size distribution for adopted raw materials ((**a**): OPC (**b**): GGBFS).

Ordinary Portland cement (OPC) (Tangshan Jidong Cement Co., Ltd., Tangshan, China) and ground granulated blast furnace slag (GGBFS) (Jinan Luxin New Building Materials Co., Ltd., Jinan, China) were used in this study. The raw materials met the standard GB/T 51003-2014 [28]. All grouting samples were prepared using tap water. The chemical compositions of OPC and GGBFS are listed in Table 1 according to the XRF technique. Additionally, the particle size distribution of raw materials was obtained using a masterizer-2000 laser particle size analyzer (Malvern Instruments Ltd., Malvern, UK) after they were dried, as shown in Figure 1. The plastic expansive agent (PEA), which is a light-yellow powder primarily composed of azodicarbonamide and minor amounts of nahcolite, was used. The relevant literature can explain the main components of this agent and how they react, including the reaction time and specific process [20,29,30]. Furthermore, the polycarboxylate superplasticizer (PCE) was selected to improve the fluidity of the grout.

### 2.2. Mixture Proportions and Preparation

Various mixture proportions were designed with different cement contents, PEA dosages, and water–binder (W/B) ratios, as shown in Table 2. The grout with 0% PEA served as the control sample. The specimens were prepared according to the Chinese standard test method of cement mortar strength (GB/T 17671-2021) [31].

### 2.3. Test Methods

PEA only plays a role in the early stage of grout shrinkage. To study the expansion mechanism of PEA, the testing method employed in this study was volumetric strain to quantify grouting deformations. Volumetric measurement was conducted by monitoring the weight of grouting samples encased within elastic membranes and immersing them in tap water.

**Figure 2:**
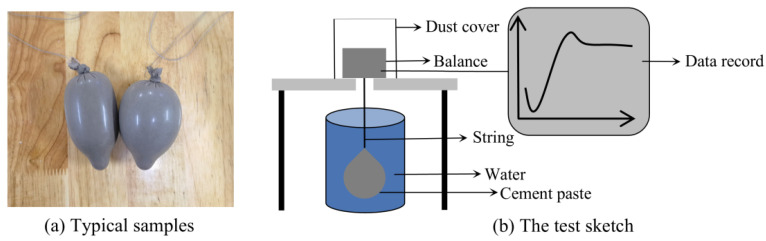
The volume expansion/shrinkage rate test.

The samples were cautiously addressed as follows: First, the grout was prepared using a cement paste mixer. Then, a total of 100–150 g of the fresh grout was poured into a polyurethane condom. The filled membrane was securely sealed with a knot, given to put out the air in the membrane. The excess part was then cut off and a string was tied to the sample [32]. Finally, the sample was put into water and the submerged weight of the samples was automatically measured and recorded at regular intervals by the controlling software. Test data were recorded using a precision balance with a precision of 0.0001 g. Measurement was recorded every 30 min starting from the moment water was added, extending up to approximately one day. Typical samples and the test sketch for the volumetric measurement of grouting samples are shown in Figure 2.

To study the relation between the PEA’s expansive process and the hydration reaction of grout, the setting time test was conducted to ascertain the initial setting time and final setting time of grouting samples employing the Vicat apparatus according to GB/T 1346-2011 [33]. Meanwhile, the pH of fresh grouts can be estimated by directly inserting a low-alkali-error glass pH electrode. The pH electrode was calibrated using pH buffer solutions of known concentrations. The compressive strength test was carried out on grouting specimens with different PEA contents according to GB/T 17671-2021. Specimens with the size of 40 mm × 40 mm × 160 mm were molded and cured at 20 ± 2 °C for 7 d, 28 d, and 56 d before the test, respectively.

To assess the impact of PEA on the air-void characteristics of hardened grouting samples using an air-void structure analyzer (Beijing Naierde Intelligent Technology Co., Ltd., Beijing, China), the specimens were cut into 100 mm × 100 mm × 10 mm ground, polished, cleaned, and dried for measurement. One surface of the sliced sample was dyed with carbon black ink. After being dyed, calcium carbonate powder was applied to smear and fill the pores. The digital images of the cross-sections of the hardened specimens were first taken, and air bubble characteristics in the test area were collected. Then, the air-void spacing factors were calculated using an area ratio method and automatically given by the measuring instrument [34]. The results were available as standard parameters for air void characterization: spacing factor (mm), specific surface (mm^2^/mm^3^), air content (%), and average air-void diameter (μm) according to GB/T 50082-2009 [35,36].

## 3. Results

### 3.1. Volume Expansion/Shrinkage Rate

**Figure 3:**
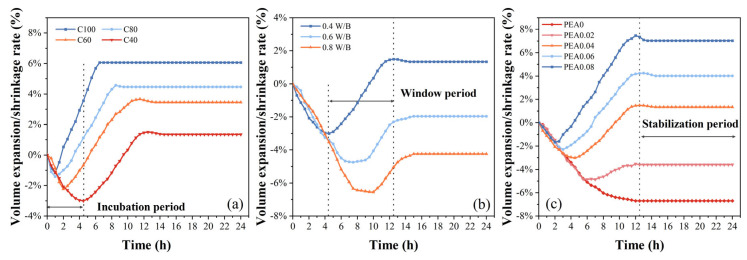
Volume expansion/shrinkage rates of grouting samples ((**a**) Cement content, (**b**) W/B ratio, (**c**) PEA content).

The results of volume expansion/shrinkage rates of grouting samples with various proportions are shown in Figure 3a–c, respectively. As seen in Figure 3a, the volume expansion rate of the grout without GGBFS was nearly 1.68% at 3 h and gradually increased to 6.06% at 24 h. The volume of the grout with 40% cement shrunk to 97.39% of its original volume at 3 h, and the volume expansion rate was nearly 1.34% at 24 h. As the cement dosage decreased, it could be seen that the volume expansion rates of specimens gradually decreased.

From Figure 3b, the sample with the 0.8 W/B ratio exhibited shrinkage behavior. The grout’s volume decreased to 97.93% of its initial volume after 3 h and continued to diminish, reaching 95.77% at 24 h. As the W/B ratios increased, the volume expansion rates of specimens gradually decreased. When the W/B ratios exceeded 0.6, the expansion effect of PEA was not effectively exerted.

The volume changes in grouts containing 0%, 0.02%, 0.04%, 0.06%, and 0.08% PEA were measured. From Figure 3c, the control grout without PEA exhibited shrinkage behavior. The volume shrinkage rate was roughly 2.63% at 3 h, stabilizing at around 6.70%. Compared to the control group, the grout with PEA showed higher volume expansion rates and variations in PEA dosages had a significant impact. As the dosage gradually increased from 0.02% to 0.08%, the grout’s volume decreased to 97.35% and 98.99% of its initial volume at 3 h, respectively. With 0.02% PEA, the grout’s volume continued to decrease, reaching 96.41% of its original volume at 24 h. However, the grout containing 0.08% PEA exhibited a volume expansion rate of nearly 7.04% at 24 h. These results implied that moderate PEA could compensate for shrinkage effectively. Based on the isotropic assumption, the volumetric strain is three times the linear strain. Sant et al. [37] showed that linear and volumetric measures of early shrinkage strain can give similar results. As the PEA content increased, the grout’s volume expansion rate also rose. The test results were compared with other research findings, demonstrating that these results were consistent with previous reports [21].

### 3.2. Setting Time

**Figure 4:**
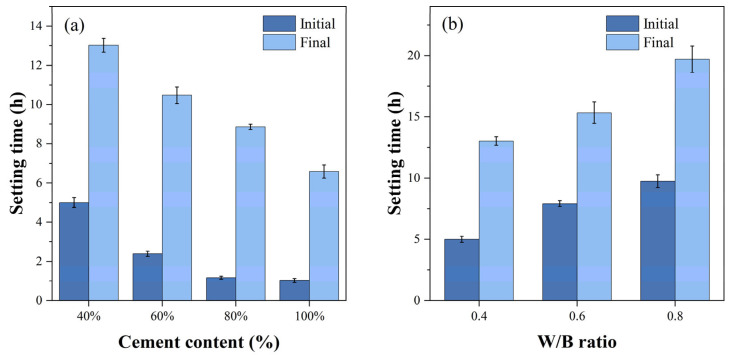
Setting times of grouting samples. (**a**) Cement content (%). (**b**) W/B ratio.

Figure 4 presents the results of initial and final setting times of grouting samples with varying different amounts of cement contents (40%, 60%, 80%, and 100%) and W/B ratios (0.4, 0.6, and 0.8). As shown in Figure 4a, with the addition of 100%, 80%, 60%, and 40% cement contents, the initial setting times of the samples were 5 h, 2.39 h, 1.17 h, and 1.03 h, respectively. The final setting times were 13.03 h, 10.47 h, 8.86 h, and 6.58 h, respectively. The setting times decreased as the cement content increased. From Figure 4b, the setting times of all grouting samples increased with increasing W/B ratios. The initial setting times of the samples changed in the range of 5–9.75 h, and the final setting times ranged from 13.03–19.69 h.

### 3.3. pH

**Figure 5:**
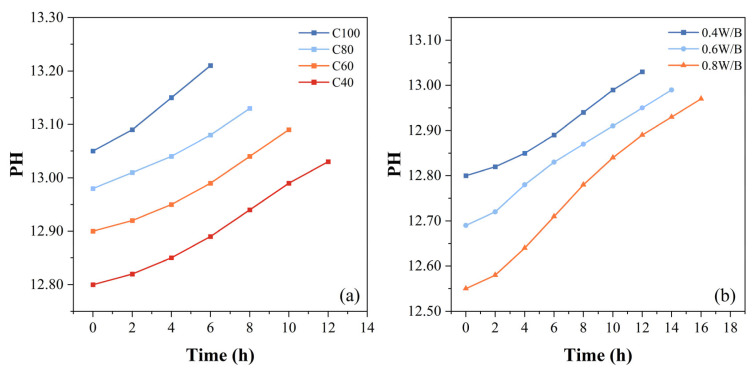
Change curve of the pH of grouting samples ((**a**) Cement content; (**b**) W/B ratio).

To further investigate the effects of mixture proportions on the expansion mechanism of PEA, the results of pH with various proportions are shown in Figure 5. The pH is an important parameter for evaluating the alkalinity level of cement-based grouting materials. The pH of fresh grouts can be estimated by directly applying the pH electrode to fresh grouting samples [38]. It could be seen that the pH gradually increased with the development of cement hydration. The pH of the 100% cement group was 13.05 at first and stabilized at 13.21 until the grout’s setting process was finished. However, the pH of the 40% cement group was 12.80 at first and slowly increased to 13.03. As the cement content increased, the pH gradually rose, and the change rate of pH gradually accelerated. The pH of the 0.8 W/B ratio group was 12.55 at first and just increased to 12.84 before the initial setting of the grout. As the W/B ratio increased, the pH gradually decreased and the rate of pH change gradually slowed down.

### 3.4. Compressive Strength

**Figure 6:**
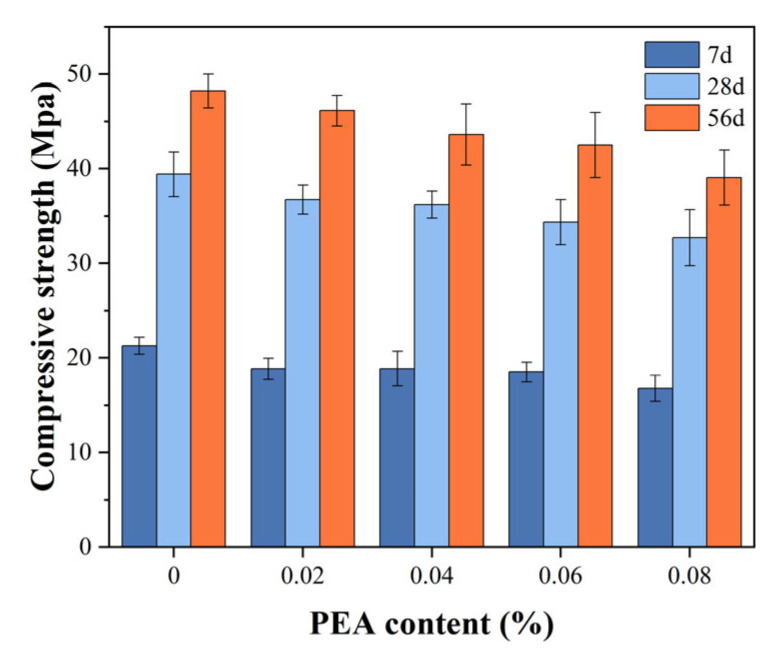
Compressive strength of grouting samples with various PEA contents.

The effects of PEA on the compressive strength of grouting samples are illustrated in Figure 6. It is obvious that the compressive strength of samples increased rapidly in the early period and was basically stable after 56 d. Specifically, the compressive strength of the 0.02%, 0.04%, 0.06%, and 0.08% PEA groups were 46.14, 43.62, 42.52, and 39.08 MPa at 56 d, respectively. These results decreased by 4.33%, 9.56%, 11.85%, and 18.98% compared to the control group. From the above results, as the content increased, PEA decreased the compressive strength of grouting samples to some extent.

### 3.5. Air-Void Parameters

The air-void parameters of grouting specimens are shown in Table 3. The results indicated that the air-void spacing factor in the case of the control group was almost three times bigger than that of the grout with 0.08% PEA. An elevation in PEA contents led to a reduction in air-void spacing factors and an augmentation of the air contents. Therefore, the air-void spacing factor serves as an effective means to delineate the impact of PEA on gas production.

**Figure 7:**
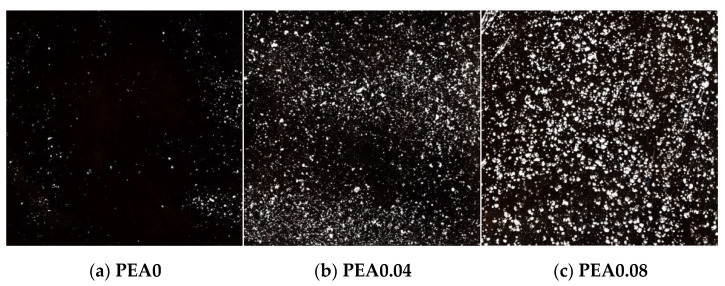
Digital images of cross-sections of hardened grouting samples with various PEA contents.

**Figure 8:**
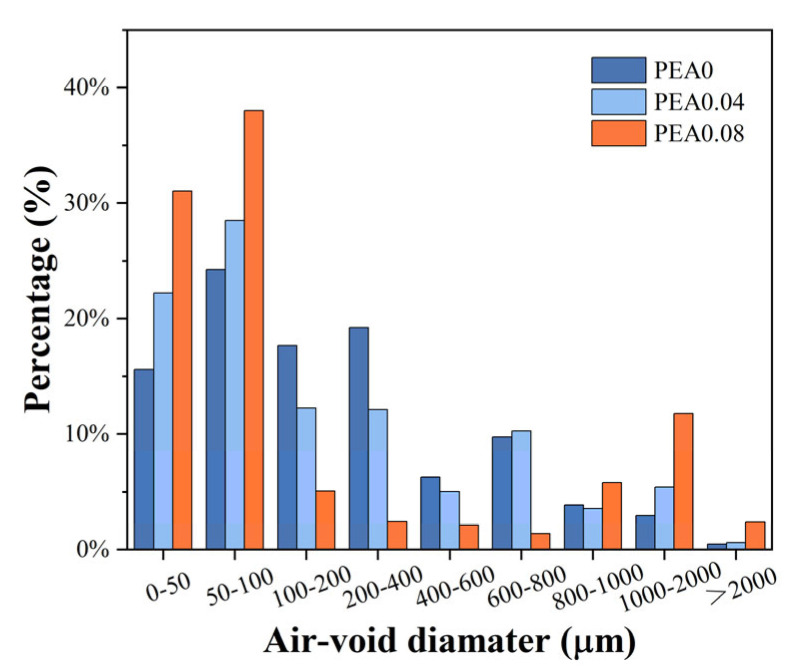
Air-void distribution of hardened grouting samples with various PEA contents.

Furthermore, digital images of cross-sections of hardened grouting samples are shown in Figure 7. The results indicated that PEA generated air bubbles with tiny size and excellent sphericity. As PEA contents rose, the number of air bubbles increased accordingly. The gas tended to generate new air bubbles in proximity to the existing ones. However, when the content of PEA was high, the existing bubbles tended to grow larger. Meanwhile, Figure 8 examines the air-void distribution. Notably, significant differences in the air-void distribution were observed with varying PEA dosages. The proportion of air-voids with areas less than 100 µm of the control group was 39.9%. However, when the PEA content reached 0.08%, there was a notable increase in air-voids smaller than 100 µm compared to the control group, while a slight increase in air-voids larger than 800 µm was observed.

## 4. Discussion

### 4.1. Mechanisms of Expansion Effect of PEA

#### 4.1.1. Expansion Process of PEA

The shrinkage of cement-based grouting materials occurs in two stages: early and later. The early stage, which occurs within the first 24 h, is when the grout is fresh and beginning to harden. The later stage refers to shrinkage that occurs after 24 h [39]. Early shrinkage significantly contributes to the risk of early cracking. Measurements of early shrinkage strain have been carried out in two fundamentally different ways: volumetric strain measurement and linear strain measurement. Compared to the linear method, one key advantage of the volumetric method lies in its capability to commence measurements immediately [40].

Based on the test results above, the expansion process of grouts containing PEA can be clearly divided into three stages. The first stage, serving as the incubation period for PEA to play its role, is characterized by an increase in the volume shrinkage rate of the grout as shown in Figure 3a. Due to varying cement contents, the end time occurred approximately 1–4.5 h after adding water and stirring. The second stage is the window period for volume expansion. At this stage, the volume expansion deformation enters a rapid increase phase, gradually reaching the peak. From Figure 3b, this stage lasted for about 4.5–12.5 h at the 0.4 W/B ratio group. The third stage is the stabilization period for volumetric change. At this stage, the volume expansion rate has fallen back to a fundamentally stable equilibrium value.

Meanwhile, the results indicated that the window period of PEA’s expansion process was correlated with the setting time as shown in Figure 4. The window period for volume expansion begins after the initial setting time of the grout, and the final setting time is basically the end time of this period. Once the grout reaches its final setting and attains a certain strength, PEA struggles to produce effective expansion.

#### 4.1.2. The Effects of PEA on Cracking Resistance

In general, the measured shrinkage encompasses a combination of autogenous shrinkage, plastic shrinkage, and chemical shrinkage [41]. During the early stage, the shrinkage of the grout primarily presents as the macroscopic volume reduction. Tricalcium silicate (C_3_S) and dicalcium silicate (C_2_S) constitute the primary components of Portland cement, but C_2_S exhibits a significantly slower hydration rate compared to C_3_S. The hydration of C_3_S dominates the hydration process, particularly during the early stage [42,43]. When the C3S mixes with water, a reaction takes place immediately, resulting in an increase in pH above 12 within a few minutes. However, this phase ends within 15 min. Afterward, the reaction rate slows down until the initial set, at which point the reaction rate begins to accelerate. The chemical reaction equation is presented as follows:

3CaO·SiO_2_ + nH_2_O ⟶ xCaO·SiO_2_·(n – 3 + x)H_2_O + (3 − x)Ca(OH)_2_ (4)

2CaO·SiO_2_ + nH_2_O ⟶ xCaO·SiO_2_·(n – 2 + x)H_2_O + (2 − x)Ca(OH)_2_ (5)

Based on the test results of pH presented above, the pH of the grout slowly increased before the initial setting and swiftly grew during the setting period, which is consistent with the mechanism of the C_3_S hydration reaction mentioned above. From Equations (1)–(3), PEA primarily reacts with the OH^−^ ions produced by the hydration of C_3_S. During the incubation period of PEA’s expansion process, the amount of Ca(OH)_2_ is limited because of the slow hydration of C_3_S. Therefore, PEA is almost non-functional, and the amount of gas generated by the PEA reaction during this state is limited. The volume shrinkage produced by the hydration reaction gradually increases, leading to a decrease in the volume of the grout during this stage.

In the window period, as C_3_S hydration progresses into the accelerated phase, a large amount of Ca(OH)_2_ is generated. The PEA particles disperse throughout the grout and gradually release gas by chemical reaction with OH^−^ ions, resulting in a rapid increase in the volume of bubbles in the grout. The increased volume of existing gas (NH_3_ and N_2_) gradually exceeds the decreased volume of shrinkage, and the volume expansion gradually reaches the peak. This peak is related to the pH of the grout. The higher the dosage of PEA, the more gas was produced, resulting in a better expansion effect in this stage, as shown in Figure 3c. In the stabilization period, the volume of grout tends to have a stable value because PEA has gradually been consumed and the grout has fully hardened.

From the analyses above, it is evident that PEA enhances the cracking resistance by generating air bubbles during the early shrinkage stage of the grout. The increased volume of these air bubbles can compensate for the decreased volume of early shrinkage and even induce slight expansion. As the PEA content increases, the grout’s volume expansion rate also rises. However, when preparing grout with PEA, it is not advisable to blindly pursue the increase in the volume expansion rate. The increase in air content can decrease the compressive strength of the cement and concrete [44]. For every 1% increase in air content, the compressive strength of the cement and concrete will decrease by 4–6% [45]. From the results in Figure 7, when the PEA content was high, the existing bubbles tended to grow larger. The gradual increase in larger air bubbles was not conducive to the compaction of the grout structure, resulting in a decrease in the compressive strength of the grout, as shown in Figure 6.

### 4.2. The Effects of Mixture Proportions on Expansion Mechanism of PEA

When the content of GGBFS is less than 70%, it gradually restrains cement hydration with its contents increasing. During the early hydration of GGBFS, it consumes Ca(OH)_2_ produced by cement hydration, which reduces the pH in the hardened grout [46]. As the W/B ratio increases, the grout’s fluidity increases on one hand, and on the other hand, the cement particles are more dispersed, leading to an increase in setting time and a slower pH change.

Therefore, the expansion mechanism of PEA is closely related to the hydration process of cement. As the cement content increases, the hydration reaction rate of the grout is accelerated, leading to an earlier initial setting time of the grout. This means that the expansion window period of PEA is advanced, and due to the earlier final setting time, the duration of this window period is shortened. Conversely, as the W/B ratio increases, the hydration reaction rate of the grout slows down. An increase in the W/B ratio leads to a delayed and extended window period. In short, the mechanism linking PEA-induced expansion to cement hydration can be concluded as follows: at the same content of PEA, a higher cement content corresponds to a higher and faster volumetric expansion, while a higher W/B ratio leads to a lower and slower volumetric expansion.

**Figure 9:**
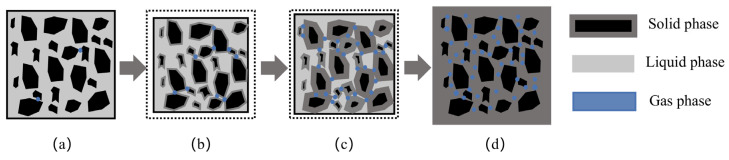
Schematic illustration of air bubbles formation and the volume change in the grout containing PEA ((**a**) Fresh grout, (**b**) Initial setting, (**c**) The initial structure largely formed; (**d**) Final setting).

Based on changes in the gas, liquid, and solid phases, the effects of mixture proportions on the expansion mechanism of PEA can be better explained by Figure 9.

Before the initial setting of the grout, the liquid phase is linked to the fresh grout. During this stage, the amount of gas generated by PEA is limited, making changes in the gas phase negligible. As shown in Figure 9b, as the hydration reaction takes place, the initial liquid phase is consumed. The solid phase begins to form its initial structure (roughly referred to as the initial setting). The PEA gradually releases gas through a chemical reaction with OH^−^ ions in the pore solution. Due to the increased viscosity of the grout, this gas can remain trapped within the grout. However, if the hydration rate is too slow, the development of the viscosity and strength is too slow, making it difficult for gas to remain trapped within the grout and form effective expansion. It could be seen that when W/B ratios exceed 0.6, the expansion effect of PEA was not effectively exerted, as shown in Figure 3b. Therefore, from the analysis above, when the W/B of the grout is high, the proportion of mineral admixtures in the mix proportion of the grout should not exceed 40%, otherwise it is difficult for PEA to produce sufficient expansion.

As the hydration reaction persists, the liquid phase is fundamentally consumed. Consequently, a large number of initial structures are formed and the number of air bubbles increases. Once the grout has fully hydrated (roughly referred to as the final setting), these air bubbles become micropores. If the hydration rate is too rapid, it can lead to an accelerated development of the grout’s setting time and strength. The occurrence of the PEA’s window period advances, resulting in the early generation of volumetric expansion. However, the higher early strength imposes greater constraints on the development of the volumetric expansion. When the cement dosage was 100%, the window period continued for about 5 h, as shown in Figure 3a, which was evidently shorter than for other proportions. Therefore, from the analysis above, when the cement dosage of the grout is high, the W/B ratio is recommended to be above 0.6 to ensure that PEA has sufficient time to exert its expansion effect.

Based on the above analyses, it is essential for effective expansion to control the window period of PEA, and it can be varied by adjusting the hydration rate of cement. The mixture proportions have a significant impact on the cement hydration of fresh grout, thereby influencing its setting time, pH, and strength development process. By adjusting the mixture proportions, the window period can be designed with cement-based grouting materials containing PEA to exhibit microexpansion properties. This research elucidated the mechanism of early shrinkage in PEA compensating grouting, but our results lack the long-term effects or performance of PEA under diverse environmental conditions and a comparison of the expansion effect between PEA with other traditional expansive agents. Further research can be conducted on these issues in the future to refine the PEA expansion theory and promote its application in the engineering field.

## 5. Conclusions

The effect of a plastic expansive agent (PEA) on the volume stability of grout was investigated in this study. The expansion effects of PEA were evaluated by the volume expansion/shrinkage rate test, and the expansion mechanism of PEA was discussed in detail according to the results of setting time, pH, compressive strength, and air-void parameters. According to the above experimental results, the following conclusions are obtained:

(1). The expansion process of PEA could be clearly divided into three stages: the incubation period, the window period, and the stabilization period. The window period was correlated with the setting time of the grout. Once the grout reaches its final setting and attains a certain strength, PEA struggles to produce effective expansion.
(2). As the content of PEA increased, the grout’s resistance to cracking improved. The expansion mechanism involves generating tiny bubbles during the grout’s early shrinkage stage. These air bubbles increase in volume, compensating for the early shrinkage and potentially causing slight expansion.
(3). The bubble spacing coefficient effectively illustrates the impact of PEA on gas production. As the PEA content increased, the bubble spacing coefficient decreased and air contents increased. However, it is not advisable to blindly increase its content. Its higher content can hinder the compaction of the grout structure, leading to a decrease in the compressive strength of the grout. Therefore, there exists an optimal dosage range for the grout containing PEA of approximately between 0.04% and 0.06%.
(4). The mixture proportions play a crucial role in influencing the hydration process of fresh grout. The length of the window period is directly tied to the setting time of the grout, with its peak being influenced by the pH level of the grout. By adjusting the mixture proportions, the window period can be effectively planned, allowing cement-based grouting materials containing PEA to exhibit microexpansion properties.

## Figures and Tables

**Table 1:** Chemical compositions of raw materials.

Composition (%)	CaO	Al_2_O_3_	SiO_2_	Fe_2_O_3_	SO_3_	MgO	K_2_O	Na_2_O
OPC ^1^	63.19	5.68	20.45	3.73	2.59	2.99	0.92	-
GGBFS ^2^	37.81	18.19	31.83	0.38	1.93	8.21	0.37	0.64

^1^ OPC: Ordinary Portland cement, ^2^ GGBFS: Ground granulated blast furnace slag.

**Table 2:** Mixture proportions of grouting specimens in this paper.

Specimen ID	OPC (%)	GGBFS (%)	PCE ^1^ (%)	PEA ^1^ (%)	W/B ^2^
C40 (0.4W/B PEA0.04)	40	60	0.3	0.04	0.4
C60	60	40	0.3	0.04	0.4
C80	80	20	0.3	0.04	0.4
C100	100	0	0.3	0.04	0.4
0.6W/B	40	60	0.3	0.04	0.6
0.8W/B	40	60	0.3	0.04	0.8
PEA0	40	60	0.3	0	0.4
PEA0.02	40	60	0.3	0.02	0.4
PEA0.06	40	60	0.3	0.06	0.4
PEA0.08	40	60	0.3	0.08	0.4

^1^ The percentage of admixture mass in the total mass of cementitious materials in the grout. ^2^ The mass ratio of water to binder.

**Table 3:** Parameters of air-void systems of grouting specimens with various PEA contents.

Specimen ID	Specific Surface Area (μm^2^/µm^3^)	Average Air-Void Diameter (µm)	Air Content (%)	Air-Void Spacing Factor (µm)
PEA0	0.051	48	0.48	271
PEA0.04	0.046	51	4.97	111
PEA0.08	0.037	50	8.35	97

## Data Availability

The original contributions presented in this study are included in the article. Further inquiries can be directed to the corresponding authors.
